# Active Metabolites as Antidepressant Drugs: The Role of Norquetiapine in the Mechanism of Action of Quetiapine in the Treatment of Mood Disorders

**DOI:** 10.3389/fpsyt.2013.00102

**Published:** 2013-09-12

**Authors:** Francisco López-Muñoz, Cecilio Álamo

**Affiliations:** ^1^Faculty of Health Sciences, Camilo José Cela University, Madrid, Spain; ^2^Department of Pharmacology, Faculty of Medicine, University of Alcalá, Madrid, Spain

**Keywords:** active metabolites, antipsychotic drugs, norquetiapine, quetiapine, mood disorders

## Abstract

Active metabolites of some antipsychotic drugs exhibit pharmacodynamic and pharmacokinetic properties that may be similar to or differ from the original compound and that can be translated by a different profile of responses and interactions to clinical level. Some of these antipsychotics’ active metabolites might participate in mechanisms of antidepressant activity, as m-chlorophenylpiperazine (aripiprazole), 9-OH-risperidone and norquetiapine. Norquetiapine exhibits distinct pharmacological activity from quetiapine and plays a fundamental role in its antidepressant efficacy. In this review, we analyze the differential pharmacological aspects between quetiapine and norquetiapine, both from the pharmacokinetic and pharmacodynamic perspectives (affinity for dopaminergic, noradrenegic, and/or serotonergic receptors, etc.), as well as differential neuroprotective role. The pharmacological differences between the two drugs could explain the differential clinical effect, as well as some differences in tolerability profile and drug interactions. The available data are sufficient to arrive at the conclusion that antidepressant activity of quetiapine is mediated, at least in part, by the active metabolite norquetiapine, which selectively inhibits noradrenaline reuptake, is a partial 5-HT_1A_ receptor agonist, and acts as an antagonist at presynaptic α_2_, 5-HT_2C_, and 5-HT_7_ receptors.

## Introduction

The body treats drugs as foreign, or xenobiotic, substances, so it tends to metabolize them to facilitate elimination. Usually, the products of biotransformation are referred to as metabolites and tend to be more water soluble than the original molecule so the kidneys can more easily eliminate them (1, 2).

Usually, biotransformation produces chemically stable metabolites that are neither pharmacologically nor toxicologically active. In some cases, though, chemically reactive metabolites are generated and may be toxic. In addition, the reactions involved in biotransformation may produce chemically stable and pharmacologically active metabolites, termed “active metabolites” (3); those will be the primary focus of this article.

Active metabolites exhibit pharmacodynamic and pharmacokinetic properties that may be similar to or differ from the original compound. In either case, they alter its activities and play a role in its end result. In fact, the presence of active metabolites is akin to polytherapy (2–4). Many medications give way to active metabolites. They are only considered clinically relevant when this occurs in high enough quantities to have a pharmacological impact, are at least as active as the original compound, or exist in specific tissues, like the brain (1, 2).

An active metabolite can be entirely or only partially responsible for a drug’s therapeutic effect. Others may be pharmacologically active in a way not related to the original molecule’s main activity. Sometimes, a metabolite reverses or deactivates the original molecule’s pharmacological activity (3). It is widely believed that many psychoactive drugs’ effects are the result not only of the original molecule, but its active metabolites. Moreover, some drugs are inactive *per se*, and must be biotransformed in the body to become pharmacologically active; these have been called prodrugs.

At times, when an active metabolite improves the original compound’s pharmacodynamic, pharmacokinetic, or toxicological properties, it gets commercialized. Examples of this in psychopharmacology include desipramine (desmethyl imipramine), derivatives of the antidepressant imipramine, mesoridazine and paliperidone, derivatives of the antipsychotics thioridazine and risperidone, and oxazepam, diazepam’s metabolite (1, 3).

## Active Metabolites of Antidepressant Drugs

Antidepressants are a wide and varied group of drugs involving many active metabolites. Some such metabolites have even been commercialized as active agents in their own right. Table 1 presents the major known active metabolites and their corresponding antidepressants.

**Table 1:** **Active metabolites of psychotropic drugs with antidepressants properties**.

Parent drug	Active metabolite	Reference
**ANTIDEPRESSANTS**		
Amitriptyline	Nortriptyline*	Owens et al. (5)
Nortriptyline	10-OH-Nortriptyline	Nordin and Bertilsson (6)
Imipramine	Desimipramine*	Owens et al. (5)
Desimipramine	Desmethyl desimipramine	Deupree et al. (7)
Trimipramine	Desmethyl trimipramine, 2-OH-Trimipramine, Trimipramine *N*-oxyde	Haenisch et al. (8)
Clomipramine	Desmethyl clomipramine^#^	Tekes et al. (2)
Doxepin	Desmethyl doxepin	Tekes et al. (2)
Mianserine	Desmethyl mianserine	Kang et al. (9)
Mirtazapine	Desmethyl mirtazapine	Sitzen and Zivkov (4), Zivkov et al. (72)
Maprotiline	Desmethyl maprotiline	Wille et al. (1)
Trazodone	*m*-Chlorophenylpiperazine	Wille et al. (1)
Citalopram	Desmethyl citalopram, Didesmethyl citalopram	Tatsumi et al. (12), Popik (13), Milne and Goa (14), Caccia (73)
Escitalopram	Desmethyl citalopram, Didesmethyl citalopram	Tatsumi et al. (12)
Fluoxetine	Desmethyl fluoxetine	Owens et al. (5), Wong et al. (16)
Fluvoxamine	Fluvoxamine acid	Benfield and Ward (15), Caccia (73)
Sertraline	Desmethyl sertraline	Fuller et al. (11), Caccia (73)
venlafaxine	*N*-Desmetil venlafaxine*	Owens et al. (5), Muth et al. (20), Caccia (73)
Atomoxetine	4-Hydroxy atomoxetine	Tekes et al. (2)
**ANTIPSYCHOTICS**		
Aripiprazole	*m*-Chlorophenylpiperazine	Caccia (33)
Risperidone	9-OH-Risperidone *^#^	Yang and Liang (38)
Quetiapine	Norquetitapine	Jensen et al. (49)

*Marketed as individual drug; ^#^suspected activity.

Nortriptyline is an active metabolite in amitriptyline and features distinctly potent inhibition of monoamine transporters, noradrenaline (NET), and serotonin (SERT) beyond that of its parent compound. Thus, while amitriptyline inhibits both transporters, its metabolite, nortriptyline, acts primarily as an inhibitor of NET reuptake (5). In turn, 10-OH-nortriptyline, considered an active metabolite of nortriptyline, is a stronger NET inhibitor and is less anticholinergic (6).

Desipramine is an active metabolite in imipramine, which inhibits reuptake of NET and SERT; the metabolite features almost selective NET inhibition. Desipramine and nortriptyline, in fact, are the strongest NET reuptake inhibitors of the tricyclic antidepressants commercially available (5). Desipramine, too, is converted into an active metabolite, desmethyl desipramine, which has more affinity for SERT than for NET. This metabolite reduces desipramine’s selectivity and adds to its overall antidepressant effect (7).

Trimipramine also involves several pharmacologically active metabolites. One is desmethyl trimipramine, which inhibits NET and SERT transporters as strongly as its parent compound. However, 2-OH-trimipramine is weaker and trimipramine-N-oxide preferentially inhibits SERT (8).

Other tricyclic antidepressants have active metabolites, but their differential profiles relative to their parent compounds have yet to be clearly elucidated. However, clomipramine is known to give way to desmethyl clomipramine, and doxepin to the metabolite desmethyl doxepin (2). Mianserin is demethylated to produce an active metabolite, *N*-desmethyl mianserin, whose longer half-life increases the effect of mianserin (9).

Mirtazapine undergoes extensive metabolism during its first pass through the liver, the major metabolic pathways in humans being N-demethylation, N-oxidation, and 8-hydroxylation, followed by conjugation. The demethylated metabolite, normirtazapine, remains pharmacologically active, but is three to four times less active than mirtazapine (4, 10).

Among selective serotonin reuptake inhibitors (SSRIs), citalopram, fluoxetine, and sertraline are initially demethylated, generating desmethyl citalopram, norfluoxetine, and norsertraline, which also inhibit SERT (11). Norcitalopram’s steady state plasma concentration approaches nearly 40% of citalopram’s, and it actively inhibits SERT three to eight times less than citalopram (12, 13). What is more, norcitalopram is converted into desmethyl citalopram, which acts as a weak SERT reuptake inhibitor (4, 14). Fluvoxamine is metabolized into multiple metabolites and the carboxylic acid derived from it has been shown to inhibit SERT weakly compared to the parent compound (4, 15).

Fluoxetine is metabolized through N-demethylation into its major active metabolite, norfluoxetine, which is comparable to fluoxetine, from pharmacological perspective (5, 16). However, norfluoxetine easily permeates the blood-brain barrier, reaches higher plasma concentrations, and has a higher half-life than fluoxetine (17, 18). In patients suffering from depression, norfluoxetine reaches concentrations comparable or even higher than fluoxetine (19), so it likely contributes considerably to the drug’s major SERT inhibition, therapeutic effectiveness, and adverse effects (4).

Sertraline gives way to a demethylated metabolite called desmethyl sertraline, which is a weaker SERT reuptake inhibitor than sertraline *in vivo*. However, it may contribute to the prolonged SERT transporter inhibition achieved by administering sertraline; laboratory research has shown this to be more the case for mice than for rats. Nevertheless, it remains unclear how much norsertraline boosts the original compound’s therapeutic activity in humans (4, 11).

Venlafaxine is a dual uptake inhibitor of SERT and NET. In humans, most venlafaxine metabolism, almost 60%, takes place through O-demethylation. Other minor metabolic routes include N-demethylation, which produces *N*-desmethyl venlafaxine. O-desmethyl venlafaxine exhibits similar pharmacological activity to that of its parent compound, both *in vitro* and *in vivo*. The derivative *N*-desmethyl is also active, but has less capacity for SERT and NET reuptake inhibition than venlafaxine does (4, 5, 20).

Atomoxetine is a selective NET reuptake inhibitor employed in the treatment of attention deficit hyperactivity disorder (ADHD). It generates a series of oxidative metabolites. Of those, 4-hydroxy-atomoxetine stands out because it blocks NET with demonstrably similar ability to atomoxetine. Atomoxetine’s remaining oxidative metabolites, including *N*-desmethyl atomoxetine, all seem to be pharmacologically inactive (21).

## Active Metabolites of Atypical Antipsychotic Drugs with Antidepressant Activity

Antipsychotics have a long history of effectiveness at treating depressive profiles. In 1954, Kielhoz suggested chlorpromazine was effective at treating depressive and manic episodes. Then Nahunek et al. (22) reported that the first atypical antipsychotic, clozapine, effectively treated endogenous depression. Today, atypical antipsychotics are recognized as effective at treating bipolar depression (23, 24), but in unipolar depression profiles, its efficacy has been mainly shown as a combined, or adjunctive, treatment in resistant profiles, an indication for which certain antipsychotics have received official approval (25, 26). The question this article aims to address is whether any of these antipsychotics’ active metabolites (see Tables 1 and 2) might participate in mechanisms of antidepressant activity.

**Table 2:** **Pharmacodynamic properties of quetiapina, risperidone, and their active metabolites (norquetiapine and paliperidone)**.

Receptors	Quetiapine*	Norquetiapine*	Risperidone**	9-OH-risperidone (paliperidone)**
D_2_	245	196	4.9	9.4
5-HT_2A_	100	48	0.17	1.9
5-HT_2C_	2502	107	12	48
5-HT_1A_	432	45	427	640
5-HT_7_	307	76	–	–
H_1_	11	3.5	5.2	5.6
Alpha-1	22	144	5	2.5
Alpha-2	3630	237	151	4.7
NET	>10000	58	–	–

Data are in Ki (nanomoles).

NET (norepinephrine transporter).

*Jensen et al. (49).

**PDPS K_i_ database: http://pdsp.med.unc.edu/pdsp.php).

Olanzapine, coupled with fluoxetine, was the first case of an atypical antipsychotic drug showing greater efficacy than each active principle separately (26, 27). Olanzapine produces various metabolites, of which conjugated 10-*N*-glucuronide has noteworthy applications in humans. Other minor metabolites include *N*-desmethyl olanzapine, 4-N-oxide-olanzapine, 2-hydroxymethyl olanzapine, and 4-*N*-glucuronide. It is noteworthy that both *in vitro* and *in vivo*, its primary metabolite, 10-*N*-glucuronide, is inactive. Of the three remaining, minor metabolites, *N*-desmethyl olanzapine and 2-hydroxymethylolanzapine display similar activity *in vitro* as their parent compound, while the rest are inactive or nearly so. These data indicate that all of olanzapine’s metabolites are less active than it, so their activity probably does not contribute to its pharmacological profile (28).

Of the available atypical antipsychotics, aripiprazole has proven effective as adjunctive treatment for major depression when it is resistant to treatment with antidepressants (29, 30). FDA has officially approved this indication. Aripiprazole’s antidepressant action may be due, first, to its role as a partial agonist of D_2_, D_3_, and 5-HT_1A_ receptors, and second, to its ability to block 5-HT_2_ receptors (29). It is a piperazine derivative and may undergo an array of metabolic processes, depending on the species. Dehydro-aripiprazole is aripiprazole’s major metabolite in humans and exhibits a similar pattern of activity at the D_2_ receptor (31). Dehydro-aripiprazole has a longer half-life than the original compound (32), suggesting it may contribute to its pharmacological efficacy. One of its metabolites, 2-3-dichlorophenylpiperazine, is an analog of m-Chlorophenylpiperazine (mCPP), an active metabolite of trazodone and nefazodone. This metabolite reaches a concentration in the brain 7% that of aripiprazole, so it could contribute only partially to that particular atypical antipsychotic’s antidepressant effect (33).

Risperidone has shown its effectiveness as adjunctive therapy, especially in conjunction with SSRIs, in patients with major depression (26). It is metabolized mainly by CYP450-2D6 (CYP2D6), generating 9-OH-risperidone, or paliperidone, through hydroxylation. When risperidone is administered, its pharmacological effects are due to the original molecule as well as its active metabolite. Considering that many patients who take risperidone orally exhibit plasma levels of 9-OH-risperidone 5–10 times higher than risperidone (34), we consider the metabolite to play an important role in the antipsychotic’s antidepressant effect (Table 2) (35). In fact, extended-release paliperidone, whether taken orally (36) or as an injection (37), has proven effective in treating schizoaffective disorder, a profile marked by a set of schizophrenia symptoms in addition to major depressive disorder. According to these clinical data, paliperidone seems to behave like a metabolite of risperidone and to have antidepressant activity. Nevertheless, to date, paliperidone has never been studied in the treatment of major depression, as monotherapy or adjunctive therapy. Yang and Liang (38) did, however, report one case where a patient with treatment-resistant depression was successfully treated with paliperidone as adjunctive therapy.

With regard to quetiapine, this particular atypical antipsychotic drug is extensively metabolized in the liver, where many of its metabolites are produced. Its most important metabolite is *N*-desalkyl quetiapine, also known as norquetiapine, which exhibits distinct pharmacological activity from quetiapine and plays a fundamental role in its antidepressant efficacy. This article will explore norquetiapine in depth in a special section below.

## Norquetiapine: Active Metabolite of Quetiapine with Antidepressant Activity

The efficacy of quetiapine in treating psychotic disorders, both schizophrenia and mania, as well as mood disorders and anxiety disorders, leads us to view it as a multifunctional psychoactive drug. Its broad spectrum of efficacy is likely due to its ability to modify systems of dopaminergic, noradrenergic, and serotonergic neurotransmission. Its effects appear to be mediated by the actions of both quetiapine and norquetiapine.

Quetiapine is a dibenzothiazepine derivative available in two different pharmaceutical forms: immediate-release (IR) and extended-release (XR) (39). Both forms are metabolized extensively by the liver into various metabolites, with only 1% eliminated unaltered, in this case in urine (40).

*N*-desalkyl quetiapine, also known as norquetiapine, is quetiapine’s key metabolite and is produced by the action of isoenzyme CYP3A4 in cytochrome P450. No genetic polymorphisms affect CYP3A4, so variations in quetiapine metabolism based on race or genetics are unlikely. However, there may be interaction at the level of this isoenzyme with some inductors (carbamazepine, phenytoin) that increase the proportion of norquetiapine, or with potent enzyme inhibitors (ketoconazole, itraconazole, erythromycin, and fluvoxamine) that slow its production (41, 42). On another note, there is more pharmacokinetic variability among older adults and patients with concomitant medications in the case of quetiapine than norquetiapine; norquetiapine levels are more stable (43). CYP2D6 also contributes to quetiapine metabolism, though to a lesser extent. This creates small quantities of 7-hydroxy quetiapine (44), whose activity has not yet been specifically determined. In addition, this isoenzyme metabolizes norquetiapine into 7-hydroxy-desalkyl-quetiapine, which is pharmacologically active (45).

According to Mauri et al. (46), quetiapine’s plasma concentrations do not seem sufficiently high to explain its action at receptors or its clinical effects, suggesting that active metabolites participate in its pharmacodynamics. However, the plasma concentrations of CNS drugs is a poor predictor of central activity because numerous other factors influence target engagement, including blood/brain barrier penetration, CNS accumulation, and receptor association/dissociation kinetics.

## Pharmacodynamic Profile of Norquetiapine *vs*. Quetiapine

Norquetiapine and quetiapine interact with the dopaminergic, noradrenergic, and serotonergic systems, but differ qualitatively and quantitatively in their respective abilities to alter those systems’ functioning. As with other atypical antipsychotics, quetiapine can block 5-HT_2A_ receptors with greater affinity than D_2_ receptors, which may explain its antipsychotic properties and lower propensity to cause extrapyramidal symptoms and hyperprolactinaemia (40). Furthermore, quetiapine shows affinity for different serotonergic and noradrenergic receptors, which may contribute to its antidepressant and anxiolytic effects (47). Also, its active metabolite, norquetiapine, is highly important in that in addition to inhibiting NET reuptake, it shows affinity for several SERT receptors (41, 42).

**Figure 1:**
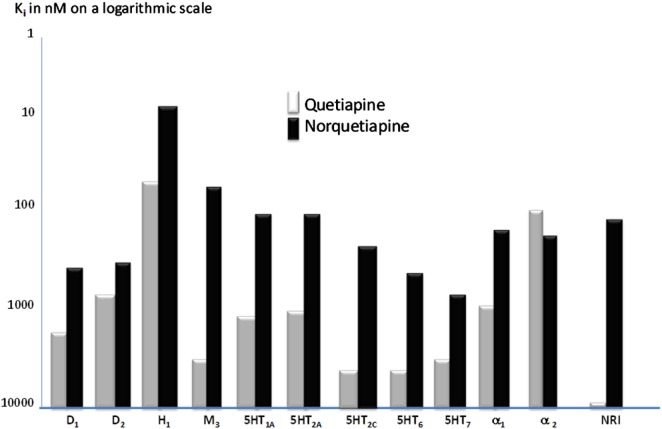
**Binding affinities of quetiapine (white bars) and its metabolite *N*-Desalkylquetiapine (dark bars) at the tested panel of brain receptors neurotransmitter transporters (*K*_i_ in nanomoles on a logarithmic scale)**. Modified to Jensen et al. (49).

An overall picture of the receptor profiles of quetiapine and norquetiapine appears in the Figure 1.

### Dopaminergic functioning

D_2_ receptor occupancy in the limbic system is the primary mechanism for bringing about antipsychotic effects. Quetiapine and norquetiapine both show moderate affinity for D_1_ and D_2_ dopamine receptor binding (*K*_i_ < 1 μM). Additionally, quetiapine dissociates rapidly from D_2_ receptors, so it must be administered at high dosages to exert an antipsychotic effect. It also exhibits good extrapyramidial tolerability and a low capacity for up-regulation at these receptors, which explains the low incidence of tardive dyskinesia associated with it (40, 48).

At the level of the nigrostriatal and tuberoinfundibular dopamine pathways, SERT seems to act as an inhibitory modulator by means of 5-HT_2A_ receptor stimulation. Quetiapine and norquetiapine are 5-HT_2A_ receptor antagonists (*K*_i_ 100 and 48 nM, respectively) (49), facilitating dopamine release along those pathways. PET studies have revealed higher 5-HT_2A_ than D_2_ antagonism along said pathways (50), which explains why quetiapine is not linked to hyperprolactinaemia and is well tolerated from an extrapyramidal standpoint. Given that norquetiapine is more adept at blocking 5-HT_2A_ than D_2_ receptors, its tolerability is similar or even better than quetiapine’s.

Moreover, greater dopamine release by quetiapine and norquetiapine not only improves its tolerability. Through various mechanisms, dopamine release in the mesocortical pathway could explain improved functioning in the prefrontal cortex (PFC) and hippocampus, which, in addition to relieving schizophrenia’s negative and cognitive symptoms, contributes to the drug’s antidepressant impact. Effectively, dopamine deficit in the PFC could explain some of the central symptoms of depression, such as anhedonia, social withdrawal, loss of motivation, and psychomotor retardation (51).

Dopamine release in the PFC is mediated by a complicated balance of several neurotransmitters and their receptors. As mentioned above, 5-HT_2A_ receptor blockage produces a spike in nigrostriatal and tuberoinfundibular dopamine levels, but also facilitates mesocortical dopamine release.

Moreover, 5-HT_2C_ receptor blockage eases cortical dopamine release (52), but that effect is secondary to inhibition of GABAergic interneurons in the brainstem (42). Thus, norquetiapine’s affinity for this receptor is low (*K*_i_ = 107 nM), but higher than its affinity for many dopamine receptors. Still, quetiapine would need concentrations 10 times higher (*K*_i_ = 2450 nM) than norquetiapine to block D_2_ receptors (49). Therefore, mesocortical dopamine release, mediated by 5-HT_2A_ blockage, is essentially due to norquetiapine.

The NET transporter is responsible for dopamine reuptake in the PFC, and norquetiapine inhibits that transporter, not quetiapine, increasing dopamine levels in the frontal cortex and adding to the antidepressant effect (49, 52). Since norquetiapine is not formed in rodents, concomitant administration of quetiapine and reboxetine, a norepinephrine reuptake inhibitor, resulted in a synergistic increase in cortical dopamine output (53).

### Serotonergic functioning

In addition to the ability of quetiapine and norquetiapine to antagonize different SERT receptors, thereby easing dopamine transmission, those agents also facilitate SERT transmission. In effect, quetiapine, and to a greater extent its metabolite norquetiapine, facilitate serotonergic transmission by behaving as partial agonists at 5-HT_1A_ receptors. 5-HT_1A_ receptors are associated with antidepressant and anxiolytic effects in humans. Presynaptic 5-HT_1A_ receptors in the raphe nuclei control serotonergic neurons’ release, while those located in postsynaptic limbic and cortical regions modulate serotonergic functioning. In fact, azapirones, like buspirone and gepirone, are partial agonists at 5-HT_1A_ receptors whose anxiolytic and antidepressant properties have been demonstrated in controlled clinical trials (54).

Norquetiapine has a high affinity for 5-HT_1A_ receptors (*K*_i_ = 45 nM), similar to that of conventional 5-HT_1A_ agonists buspirone and gepirone (55) and 10 times greater than quetiapine’s (*K*_i_ = 430 nM). Moreover, in functional studies of GTPg-35s, the metabolite was also more efficacious than quetiapine itself (75 *vs*. 47% of maximum SERT response) (49).

Hence, norquetiapine can bring about activation at postsynaptic 5-HT_1A_ receptors in the hippocampus (56), an area of the brain altered by depression. This could spur neuron regeneration in that area by increasing the release of nerve growth factors like brain-derived neurotrophic factor (BDNF) (57). Also, 5-HT_1A_ receptor stimulation increases acetylcholine release in the PFC, promoting synaptic plasticity linked to learning and memory (58). Norquetiapine’s effect as a partial agonist of 5-HT_1A_ receptors may contribute, at least in part, to quetiapine’s clinically demonstrated antidepressant and anxiolytic action (59).

Norquetiapine also has a higher affinity for the 5-HT_7_ receptor (*K*_i_ = 76 nM) than quetiapine (*K*_i_ = 307 nM). This receptor’s involvement in depression and sleep-related, circadian rhythm disorders has been experimentally documented (60). While no data are available about 5-HT_7_ antagonists’ effects in humans, it is possible that norquetiapine’s 5-HT_7_ receptor antagonism contributes to quetiapine’s antidepressant action (49).

As discussed above, quetiapine, and to a greater extent norquetiapine, have antagonistic properties at 5-HT_2A_ receptors. Use of 5-HT_2A_ antagonists has been proposed to treat insomnia, so perhaps norquetiapine, which is also a potent H_1_ receptor antagonist, contributes to the highly sedative effects observed during quetiapine treatment (49).

Norquetiapine has a high affinity (*K*_i_ = 14 nM) for 5-HT_2B_ receptors (49). It is well-known that drugs that stimulate that particular receptor are closely tied to acute heart valve disease (61). However, norquetiapine binds to said receptor purely as an antagonist, not as an agonist, ruling out the possibility that it would cause valvular disease (49).

Some researchers have postulated that 5-HT_2C_ receptor blockage, paired with H_1_ histamine receptor blockage, is linked to weight gain caused by treatment with antipsychotics (62, 63). Since norquetiapine binds to 5-HT_2C_ receptors (*K*_i_ = 107 nM) with much more potency than quetiapine (*K*_i_ = 2502 nM), while also blocking histamine receptors (*K*_i_ = 3.5 nM), weight gain may be primarily the fault of this metabolite (49).

### Noradrenergic functioning

Norquetiapine acts as a potent NET transporter inhibitor (*K*_i_ = 23 nM), while quetiapine does not actively inhibit said transporter. Norquetiapine’s affinity for the NE transporter (*K*_i_ = 58 nM) is similar to that of certain other antidepressants like nortriptyline, amitriptyline, and duloxetine (49). Quetiapine, in contrast, cannot bind to the NET transporter. This differential feature of norquetiapine *versus* quetiapine may have an impact on antidepressant ability.

Also, norquetiapine increases noradrenergic functioning by blocking presynaptic α_2_ receptors (*K*_i_ = 237 nM) considerably more than quetiapine (*K*_i_ = 3630 nM) (49). 5-HT_2A_ and 5-HT_2C_ antagonism promotes this increase in NET, which in turn raises dopamine and NET levels in the PFC, as mentioned above (42, 49, 64, 65). By inhibiting the NET transporter, norquetiapine increases dopamine levels in the PFC, because dopamine also employs the NET transporter for reuptake (49, 52).

According to data collected *in vivo*, norquetiapine at dosages as low as 0.1 mg/Kg displayed antidepressant activity in the tail suspension test in mice (49).

### Other mechanisms involved in the antidepressant effect of quetiapine

Other alternative, much less thoroughly studied mechanisms than those involving monoamines may be at work in quetiapine’s antidepressant action. For example, stress and depression are known to increase neurodegeneration, and antidepressants may counteract that effect or undo it altogether. In experimental studies, quetiapine has been shown to prevent BDNF reduction as well as chronic stress-related cellular degeneration in the hippocampus. It has been postulate that these effects could improve some of the symptoms and cognitive deficits implicated in depression (66).

Additionally, quetiapine has been observed to modulate the activity of glutamate receptors to hypothetically restore them to normal functioning. This would decrease the neurotoxicity caused by an excess of the neurotransmitter glutamate (67).

Other authors have postulated that quetiapine may exert antidepressant effects by controlling inflammatory cytokines, cytoprotective molecules, and the antioxidant effect, all of which are mechanisms associated with the onset of depressive symptomatology (68), though there is scarcely any experimental or clinical support for that hypothesis. Norquetiapine’s role in those mechanisms has not yet been studied.

### Norquetiapine’s receptor binding to blame for adverse effects

The receptor profiles of quetiapine and norquetiapine may explain the adverse effects that often arise in treating depression. The ability of quetiapine (*K*_i_ = 11 nM) and norquetiapine (*K*_i_ = 3.5 nM) to bind to H_1_ histamine receptors is associated with sedation, hypnotic effects, increased appetite, and weight gain.

Blockage of α_1_ adrenergic receptors, which both quetiapine and norquetiapine exhibit, is linked to orthostatic hypertension in some patients. This is less true of quetiapine XR, which has fewer plasmatic “peaks.”

Norquetiapine shows affinity for muscarine receptors, always acting as an antagonist, especially at subtypes M_1_, M_3_, and M_5_. This antagonism is 20–80 times stronger than what was observed with quetiapine, so it is believed that adverse atropinic effects like dry mouth, urine retention, mydriasis, increased intraocular pressure, and hypothermia result from quetiapine’s active metabolite. On the other hand, some researchers postulate that M_3_ receptor antagonism is responsible, at least in part, for the onset of hyperglycemia and diabetes caused by antipsychotic treatment (69). It would seem that most of these adverse effects following quetiapine overdose (70) are due primarily to its active metabolite, norquetiapine (49).

## Conclusion

Quetiapine is a multifunctional drug endowed with antidepressant properties through various mechanisms: noradrenergic, serotonergic, and dopaminergic. Quetiapine displays clinically significant efficacy in treating unipolar depression, both as a monotherapy (off-label use; only in Australia and Canada is this an officially approved indication) and as adjunctive therapy with antidepressants in patients who respond unsatisfactorily or not at all to antidepressants alone. Furthermore, quetiapine is the only drug demonstrated to be effective at treating unipolar and bipolar depression with no associated risk of veering into mania (68, 71).

The available data are sufficient to arrive at the conclusion that quetiapine’s antidepressant activity is mediated, at least in part, by the active metabolite norquetiapine, which selectively inhibits NET reuptake, is a partial 5-HT_1A_ receptor agonist, and acts as an antagonist at presynaptic α_2_, 5-HT_2C_, and 5-HT_7_ receptors. Furthermore, its active metabolite contributes to quetiapine’s profile of undesirable effects because norquetiapine binds at H_1_ histamine, α_1_ adrenergic, and muscarine receptors. Norquetiapine’s activity profile is shared by no other known antipsychotic or antidepressant drug (49).

Norquetiapine clearly exemplifies the case of an active metabolite that, through metabolism, can make a drug originally introduced as an antipsychotic, in this case quetiapine, become a useful antidepressant agent.

## Conflict of Interest Statement

The authors declare that the research was conducted in the absence of any commercial or financial relationships that could be construed as a potential conflict of interest.
